# Dimensional distribution of cortical abnormality across antipsychotics treatment-resistant and responsive schizophrenia

**DOI:** 10.1016/j.nicl.2021.102852

**Published:** 2021-10-07

**Authors:** Takashi Itahashi, Yoshihiro Noda, Yusuke Iwata, Ryosuke Tarumi, Sakiko Tsugawa, Eric Plitman, Shiori Honda, Fernando Caravaggio, Julia Kim, Karin Matsushita, Philip Gerretsen, Hiroyuki Uchida, Gary Remington, Masaru Mimura, Yuta Y. Aoki, Ariel Graff-Guerrero, Shinichiro Nakajima

**Affiliations:** a Medical Institute of Developmental Disabilities Research, Showa University, Tokyo, Japan; b Department of Neuropsychiatry, Keio University School of Medicine, Tokyo, Japan; c Department of Neuropsychiatry, University of Yamanashi Faculty of Medicine, Yamanashi, Japan; d Brain Health Imaging Centre, Centre for Addiction and Mental Health (CAMH), Toronto, Ontario, Canada; e Department of Psychiatry, University of Toronto, Toronto, Ontario, Canada

**Keywords:** Classification, Cortical thickness, Schizophrenia, Treatment-resistant

## Abstract

**Background:**

One-third of patients with schizophrenia are treatment-resistant to non-clozapine antipsychotics (TRS), while the rest respond (NTRS). Examining whether TRS and NTRS represent different pathophysiologies is an important step toward precision medicine.

**Methods:**

Focusing on cortical thickness (CT), we analyzed international multi-site cross-sectional datasets of magnetic resonance imaging comprising 110 patients with schizophrenia (NTRS = 46, TRS = 64) and 52 healthy controls (HCs). We utilized a logistic regression with L1-norm regularization to find brain regions related to either NTRS or TRS. We conducted nested 10-fold cross-validation and computed the accuracy and area under the curve (AUC). Then, we applied the NTRS classifier to patients with TRS, and vice versa.

**Results:**

Patients with NTRS and TRS were classified from HCs with 65% and 78% accuracies and with the AUC of 0.69 and 0.85 (*p* = 0.014 and < 0.001, corrected), respectively. The left planum temporale (PT) and left anterior insula/inferior frontal gyrus (IFG) contributed to both NTRS and TRS classifiers. The left supramarginal gyrus only contributed to NTRS and right superior temporal sulcus and right lateral orbitofrontal cortex only to the TRS. The NTRS classifiers successfully distinguished those with TRS from HCs with the AUC of 0.78 (*p* < 0.001), while the TRS classifiers classified those with NTRS from HCs with the AUC of 0.69 (*p* = 0.015).

**Conclusion:**

Both NTRS and TRS could be distinguished from HCs on the basis of CT. The CT pathological basis of NTRS and TRS has commonalities, and TRS presents unique CT features.

## Highlights

• Different etiology is assumed in treatment-resistant and responsive schizophrenia.
• Patients with treatment-resistant schizophrenia were classified from controls.
• Patients with non-treatment-resistant schizophrenia were classified from controls.
• Two classifications reached area under the curve as high as 0.69 and 0.85.
• Area under the curve remained as high as 0.69 when two classifiers were swapped.

## 1 Introduction

Schizophrenia is a severe mental disease characterized by positive, negative, and cognitive symptoms (Howes and Murray, 2014). The prevalence is about 1% of the general population across the world (Whiteford et al., 2013). Among several hypotheses about its pathology, the aberrant dopamine system is prominent (Kapur et al., 2000). Indeed, the mainstay of pharmacological treatment to the disease has been antagonists of the dopamine D2 receptor since their first appearance in the 1950s. However, a third of patients with schizophrenia do not respond well to dopamine D2 receptor antagonists. Such a condition is called treatment-resistant schizophrenia (TRS) (Beck et al., 2014). A dearth of responses to blocking dopamine D2 receptors in those with TRS suggests different etiologies between TRS and antipsychotic responders (non-TRS, henceforth, NTRS) under the same diagnosis umbrella with similar symptom patterns.

A potential etiology for the TRS involves the dysfunction of the glutamatergic system, which leads to abnormality in the GABAergic system through the N-methyl-D-aspartate (NMDA) receptors. Indeed, the only approved drug for TRS is clozapine, which has a lower affinity to the D2 receptor and higher affinity to the GABA receptor. To examine the glutamate hypothesis in TRS, prior neuroimaging studies used proton magnetic resonance spectroscopy (^1^H-MRS) to measure glutamatergic neurometabolite levels in patients with TRS (Goldstein et al., 2015, Mouchlianitis et al., 2016). Although the degree varied (Goldstein et al., 2015, Mouchlianitis et al., 2016, Demjaha et al., 2014, Marsman et al., 2013, Tarumi et al., 2020, Iwata et al., 2019), the results of these studies corroborated abnormalities in the glutamate system of those with TRS.

Structural neuroimaging studies focusing on cortical thickness (CT) contrasted patients with schizophrenia against healthy controls (HCs). These studies consistently showed thinner CT in a wide variety of brain regions in patients with schizophrenia compared with HCs (van Erp et al., 2018). More specifically, these brain regions included the insula, frontal lobe, and superior temporal region (van Erp et al., 2018). Associations between CT in these brain regions and the severity of positive symptoms indicate that CT would be a promising biological measure to schizophrenia pathophysiology (Palaniyappan et al., 2012).

Some structural neuroimaging studies contrasted TRS against NTRS (Nakajima et al., 2015). Zugman et al., compared CT among the NTRS, TRS, and HC groups (Zugman et al., 2013). They revealed that patients with TRS presented with thinner CT in widespread brain regions compared with patients with NTRS. They also showed diffusely thinner CT in patients with NTRS in comparison with HCs. Despite the distinct pattern in response to treatment, both of these two conditions showed diffusely thin CT. These findings raise the question of whether the two conditions have distinct pathophysiology at the CT level or hold similar CT pattern with a different range of deviation. The former suggests that two conditions derive from two distinct etiologies and should be clinically treated differently. On the other hand, the latter implies that both conditions are combinations of different etiologies, i.e., heterogeneous, but merge at CT level to form one continuous entity and can be clinically treated in a dimensional fashion.

The current study is motivated to address the aforementioned question. To this end, we first constructed two sets of brain regions serving as classifiers: one distinguished NTRS patients from HCs, and the other distinguished those with TRS from HCs. The built classifiers for NTRS were applied to patients with TRS and HCs to evaluate whether the NTRS classifiers can differentiate patients with TRS from HCs. At the same time, we also applied the TRS classifiers to the patients with NTRS and HCs. By exchanging these classifiers, we aimed to examine whether TRS and NTRS have different CT patterns or not.

## 2 Materials and methods

### 2.1 Participants

We used international multi-site cross-sectional neuroimaging datasets comprising 120 patients with schizophrenia and 54 HCs. Fifty-one patients (NTRS: *n* = 29, and TRS: *n* = 22) and 28 HCs were enrolled and scanned at Komagino Hospital, Tokyo, Japan, while 69 patients (NTRS: *n* = 19 and TRS: *n* = 50) and 26 HCs were enrolled and scanned at the Centre for Addiction and Mental Health (CAMH), Toronto, Canada. These original studies were approved by the ethics committees at each site (Tarumi et al., 2020, Iwata et al., 2019). All the participants were enrolled following the completion of an informed consent procedure and provided written assent. There was an overlap in participants of the current study and our previous studies (Tarumi et al., 2020, Kim et al., 2020, Ochi et al., 2020, Shah et al., 2020). After visual inspection and quality control, twelve participants were excluded from subsequent analyses due to poor imaging quality (*n* = 9) or brain anomalies (*n* = 3). Table 1 shows the demographic information of the final sample.

**Table 1:** Demographic and clinical information of participants in the dataset pooled across two sites.

	Pooled dataset			Statistics		
	HC	NTRS	TRS	Statistics	*df*	*P*-values
N (Female)	52 (23)	46 (21)	64 (20)	1.5		0.46
Age, year (mean ± SD)	41.5 ± 12.3	43.3 ± 13.2	42.8 ± 12.2	*F* = 0.27	2, 159	0.77
Duration of illness, year	–	17.5 ± 12.2	18.6 ± 11.7	*t* = −0.5	103	0.61
PANSS total score	–	52.7 ± 13.2	83.7 ± 25.4	*t* = −7.5	103	< 0.001
Positive symptom subscale	–	10.3 ± 2.7	20.5 ± 7.2	*t* = −9.0	103	< 0.001
Negative symptom subscale	–	15.0 ± 5.3	22.6 ± 7.4	*t* = −5.9	103	< 0.001
General psychopathology subscale	–	27.4 ± 6.5	40.7 ± 12.7	*t* = −6.4	103	< 0.001
CGI-S	–	2.1 ± 0.7	3.3 ± 1.4	*t* = −5.3	102	< 0.001
CPZ equivalent daily dose, (mg)	–	407.4 ± 208.2	686.6 ± 404.1	*t* = −4.3	107	< 0.001

**Abbreviations:** CGI-S: Clinical Global Impression Severity Scale, CPZ: chlorpromazine, HC: healthy control, NTRS: non-treatment-resistant schizophrenia, PANSS: Positive and Negative Symptom Scale, SD: standard deviation, TRS: treatment-resistant schizophrenia.

### 2.2 Clinical assessments

The details of inclusion criteria and clinical assessments were described in our previous studies  (Tarumi et al., 2020, Kim et al., 2020, Ochi et al., 2020, Shah et al., 2020). Patients had a diagnosis of schizophrenia or schizoaffective disorder based on the DSM-IV. We assessed symptom severity with the Positive and Negative Syndrome Scale (PANSS) (Kay et al., 1987) and Clinical Global Impression Severity Scale (CGI-S) (Guy, 1976). Antipsychotic treatment resistance was defined by the modified Treatment Response and Resistance in Psychosis (TRRIP) Working Group Consensus criteria (Howes et al., 2017). Treatment response was defined by (i) CGI-S score ≤ 3, (ii) scores of all positive symptom items of the PANSS ≤ 3, and (iii) no symptomatic relapse in the previous 3 months. In contrast, inadequate treatment response was defined by (i) CGI-S score ≥ 4 and (ii) ≥ 4 on at least 2 PANSS positive symptom items after adequate antipsychotic trials. Response to past antipsychotic trials was determined based on medical records.

HCs were assessed by the Mini-International Neuropsychiatric Interview (MINI) (Sheehan et al., 1998) to confirm if they had no history of psychiatric illness. Exclusion criteria for all study participants included: (i) substance abuse or dependence within the past six months; (ii) a positive urine drug screen at inclusion or before MRI scan; (iii) a history of head trauma resulting in loss of consciousness for over 30 min; or (iv) an unstable physical illness or neurological disorder.

### 2.3 MRI data acquisition

Similar scanning parameters were used at the two sites. At the Komagino Hospital, T1-weighted images were collected using a 3 T Signa HDxt scanner (GE Healthcare) with an eight-channel head coil (BRAVO, echo time [TE] = 2.8 ms, repetition time [TR] = 6.4 ms, inversion time [TI] = 650 ms, flip angle = 8°, field of view [FOV] = 230 mm, matrix size = 256 × 256, slice thickness = 0.9 mm). At the CAMH, T1-weighted images were collected using a 3 T GE Discovery R750 scanner (GE Healthcare) with an eight-channel head coil (BRAVO, echo time [TE] = 3 ms, repetition time [TR] = 6.74 ms, inversion time [TI] = 650 ms, flip angle = 8°, FOV = 230 mm, matrix size = 256 × 256, slice thickness = 0.9 mm).

### 2.4 Structural MRI preprocessing

All the MRI data were preprocessed using FreeSurfer version 6.0.1. The details of preprocessing steps were described elsewhere (Dale et al., 1999, Fischl et al., 1998). Briefly, this software performed a series of preprocessing steps, including spatial normalization, bias field correction, intensity normalization, skull-stripping, segmentation, and reconstruction of surface mesh. We computed CT as a representative cortical parameter in this study. We used Schaefer’s 400 cortical parcels (Schaefer et al., 2018) as an atlas to define regions of interest (ROIs) and extracted the structural characteristics from each participant.

### 2.5 Across sites harmonization

We used a ComBat harmonization method (Fortin et al., 2018; Johnson et al., 2007), to control for the site differences in the cortical parameters. The ComBat harmonization method estimates and removes the site bias while retaining biological factors (e.g., age, disease status, and sex). In this study, we performed harmonization to correct only for the site difference while considering disease status, age, sex, and years of education as biological variables in the ComBat. Of note, we coded the disease status as three groups (i.e., HC, NTRS, and TRS groups), instead of two groups (i.e., HC and SCZ groups) to avoid information leakage.

### 2.6 Constructions of classifiers

**Fig. 1:**
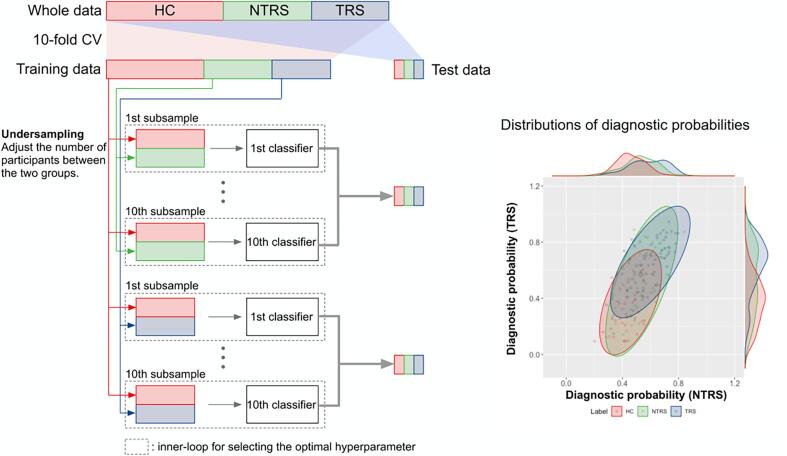
Schematic representation of the analytical procedures used in this study. The classifiers for non-treatment resistant schizophrenia (NTRS) and treatment-resistant schizophrenia (TRS) were constructed using nested cross-validation (CV). To adjust the number of samples between the groups in each loop, we also employed undersampling. The undersampling procedure was applied ten times in each loop, resulting in 10 classifiers for NTRS and TRS, respectively. To compute the diagnostic probability and the classification performance, the trained classifiers were applied to test data. Abbreviations: HC: healthy control.

We constructed two sets of brain regions serving as classifiers using 400 CT values: one for distinguishing patients with NTRS from HCs and the other for classifying those with TRS from HCs. Similar to previous studies (Yamagata et al., 2018, Yamashita et al., 2020), we employed a logistic regression with the least absolute shrinkage and selection operator (LASSO) method (Tibshirani, 1996) as a classifier model to find the optimal subset of ROIs associated with either NTRS or TRS. The optimal weight vector was determined by minimizing the following objective function:

$$J\left(w\right)=-\frac{1}{n}\sum_{i=1}^{n}loglog\left(P_{i}\left(y_{i}=1|x_{i};w\right)\right)+\lambda {\left\|w\right\|}_{1},$$

where the diagnostic probability, *P*, was defined as a logistic function. The LASSO method controls the amount of shrinkage applied to the estimates using the hyperparameter; λ. To estimate weights of logistic regression and a hyperparameter, we conducted a nested 10-fold cross-validation (CV) procedure (Fig. 1. In this procedure, we first divided the whole dataset into ten folds so that the number of participants in each group would be the same across folds as possible. Each fold contained approximately five HCs, six patients with TRS, and four those with NTRS. We then considered 9-folds out of 10 as the training dataset and the remaining fold as a test dataset for testing the trained model. We further subdivided the training dataset into two datasets: one was used for training a classifier for NTRS, and the other was used for training a classifier for TRS. The proportions of patients were imbalanced when combining the dataset from the two sites (i.e., Komagino and CAMH). We, thus, used an undersampling method based on a previous study (Yamashita et al., 2020) to minimize bias due to the imbalance in the number of participants between the two groups (e.g.; HCs and NTRS). For the inner loop, we used the “*lassoglm*” function implemented in MATLAB (R2020b, Mathworks, USA). In this function, we set “NumLambda” to 25 and “CV” to 10. In the inner loop, this function first computes a value of λ that is just large enough such that the only optimal solution is an all-zero vector. This function creates a total of 25 equally spaced λ values from 0 to λ_max_, and determines the optimal λ according to the one-standard-error rule (Friedman et al., 2001); in which this function selects the largest λ within the standard deviation of minimum prediction error among all λ. Since only a subset of training data was used after undersampling, we repeated the random sampling procedure ten times (i.e., subsampling). We then fitted a model to each subsampled training data while tuning a hyperparameter in the inner loop of the nested-CV. These procedures yielded ten classifiers in each loop. Instead of selecting a single winning model, these classifiers were applied to the test dataset. Then, the mean classifier output value (diagnostic probability) was computed. Of note, our analytical procedure is conceptually equal to the bagging technique that improves the stability and performance of machine learning algorithms (Breiman, 1996). We expected that the combination of the undersampling method with the soft-voting procedure could improve the classification performance in our analyses. We considered participants as either NTRS or TRS if their diagnostic probability values were greater than 0.5. We aggregated the predicted labels across a 10-fold CV and then computed the area under the curve (AUC) as an index for classification performance. We also computed the accuracy, sensitivity, and specificity.

A permutation test with 1,000 iterations was performed to test the statistical significance of the classification performance. We permuted the labels of the training dataset and conducted a 10-fold CV with a 10-subsampling procedure. We computed the mean diagnostic probability obtained from permuted classifiers at each iteration. We considered participants as either NTRS or TRS if their mean diagnostic probability values were higher than 0.5. We then computed the AUC values for each classification problem (i.e., HCs versus NTRS and HCs versus TRS) at each iteration. To control for the multiple comparisons, we constructed a null distribution as the max distribution of the AUC values among the two permuted classifiers. Statistical significance was set at *p* < 0.05, one-sided. We performed these analyses using MATLAB (R2020b, Mathworks, USA).

### 2.7 Identification of brain regions contributing to classifications

We identified brain regions that contributed to classifications. Given the notion that important brain regions were frequently selected by the LASSO method, we considered a brain region as important if the number of times selected by the LASSO method during 10-fold CV was statistically higher than chance. To construct a null distribution, we performed a permutation test with 1,000 iterations. In each iteration, we shuffled the diagnostic labels of the dataset and conducted a 10-fold CV with a 10-subsampling procedure. This procedure yielded 100 classifiers (i.e., 10 folds × 10 subsampling) for either NTRS or TRS at each iteration. This suggests that the maximum number of selected times is 100. We counted the number of times selected by the LASSO method in each brain region at each iteration. We then constructed a null distribution as the max distribution of the number of times among the two permuted classifiers to control the multiple comparison problem. Statistical significance was set at *p* < 0.05, one-sided.

## 3 Results

### 3.1 Classifiers for NTRS and TRS

**Fig. 2:**
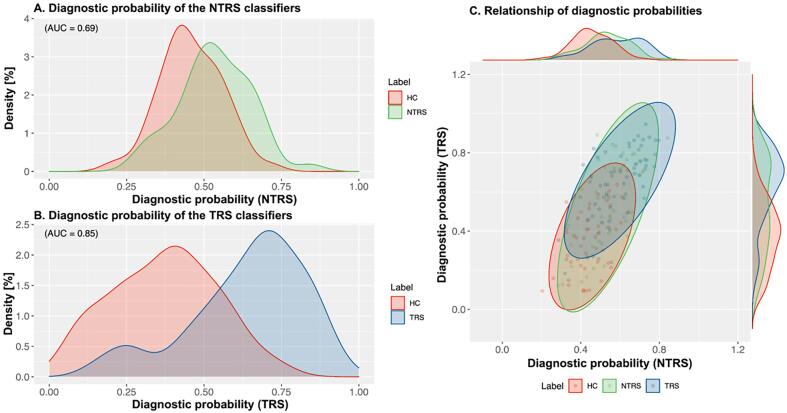
The classification performance of the classifiers for NTRS and TRS. (A) Diagnostic probability distribution for non-treatment-resistant schizophrenia (NTRS) and (B) diagnostic probability distribution for treatment-resistant schizophrenia (TRS). We computed the area under the curve (AUC). Permutation tests with 1,000 iterations were conducted to examine the statistical significance. Both classifiers successfully distinguished either NTRS or TRS from healthy controls (HCs) (all *P* < 0.05, corrected). We applied the constructed classifier for NTRS to patients with TRS while applying the classifier for TRS to those with NTRS.

We constructed two sets of neuroanatomical feature-based classifiers for NTRS and TRS, which distinguished between HCs and patients with NTRS, and between HCs and patients with TRS, respectively. The set of classifiers distinguished patients with NTRS from HCs with an accuracy of 65% and an AUC of 0.69 (*p* = 0.014, corrected) (Fig. 2A. Sensitivity and specificity were 67% and 63%, respectively. The other set of classifiers distinguished patients with TRS from HCs with an accuracy of 78% and an AUC of 0.85 (*p* < 0.001, corrected) (Fig. 2B. Sensitivity and specificity were 77% and 80%, respectively. These results suggest that both sets of classifiers successfully discriminated between each patient group (NTRS or TRS) and HCs.

To investigate the exchangeability of the set of classifiers, we applied the classifiers for NTRS to patients with TRS, and vice versa (Fig. 2C. For the classifiers for NTRS, the AUC value for distinguishing patients with TRS from HCs was 0.78 (*p* < 0.001, corrected). The classifiers for TRS distinguished patients with NTRS from HCs with 0.69 (*p* = 0.015, corrected). These *P*-values were computed using the max distribution derived by the permutation test. These results suggest that both sets of classifiers are exchangeable, and thus, the TRS and NTRS groups may be part of a continuous entity.

### 3.2 Brain regions contributing to classifications

**Fig. 3:**
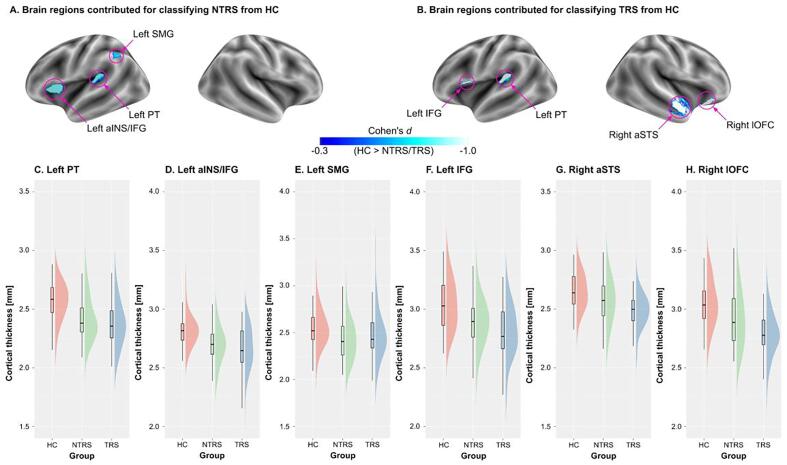
Brain regions contributed to the classifications. (A) Brain regions significantly contributed to the classification of non-treatment resistant schizophrenia (NTRS) and (B) brain regions significantly contributed to the classification of treatment-resistant schizophrenia (TRS). Distributions of cortical thickness (CT) values of each group in the left planum temporale (PT) (C), left anterior insula/inferior frontal gyrus (aINS/IFG) (D), left supramarginal gyrus (SMG) (E), left IFG (F), right anterior superior temporal sulcus (aSTS) (G), and right lateral orbitofrontal cortex (lOFC). *: *p* < 0.05, **: *p* < 0.01, ***: *p* < 0.005, and ****: *p* < 0.001.

To investigate the extent to which brain regions contributed to the classification, we counted the number of times selected during the 10-fold CV. Once important brain regions were identified using the permutation test described above, we computed Cohen’s *d* to characterize the difference between the groups. As shown in Fig. 3, three brain regions contributed to the NTRS classification: the left planum temporale (PT), left anterior insula/inferior frontal gyrus (aINS/IFG), and left supramarginal gyrus (SMG). On the other hand, four brain regions contributed to the TRS classification: the left PT, left IFG, right anterior superior temporal sulcus (aSTS), and right lateral orbitofrontal cortex (lOFC). The left PT was the only region shared by the two classifiers.

To confirm the directionality of group differences in brain regions identified by classification analyses, we computed the standardized effect size estimates (Cohen’s *d*). The analysis showed that both NTRS and TRS groups exhibited lower CT values in these brain regions than the HC group (all *p* < 0.05), except for the left SMG (NTRS: *p* = 0.01 and TRS: *p* = 0.15). The TRS group exhibited lower CT values only in the right aSTS and right lOFC than the NTRS group (*p* < 0.05).

## 4 Discussion

In the current study, we analyzed CT values and constructed two sets of classifiers for NTRS and TRS, respectively. Applying the NTRS classifier to patients with TRS, the analyses successfully differentiated the TRS group from the HC group. The TRS classifier also successfully classified the NTRS group from the HC group. While the NTRS and TRS classifiers include the contribution of various common brain regions, the TRS classifiers have two unique brain regions (right aSTS and right IOFC). The common brain regions in both sets of classifiers suggest that TRS and NTRS share neurobiological mechanisms. However, the fact that TRS classifiers includes two different regions may indicate: TRS is a more severe form of NTRS, therefore a continuum, or TRS is a different entity with some common neurobiological features with NTRS, or the differences are driven by different treatment (clozapine versus first-line antipsychotic) at the time of scanning. Given that two categorically different etiologies, namely glutamate- and dopamine-driven, are supposed in TRS and NTRS, the potential that TRS and NTRS form one continuum entity and reflect heterogeneity in the entity has significance in both clinical and scientific senses. As the current findings indicate that patients with NTRS have traits of patients with not only NTRS but TRS, a novel treatment strategy targeting glutamatergic abnormality is needed to improve the outcome in patients with NTRS. Besides, the current results call for the paradigm shift from categorically contrasting TRS against NTRS in order to investigate the neural bases of resistance to antipsychotics to assuming the dimensional and overlapping pathophysiology in both conditions.

As is the case with other studies with patients with TRS, participants of the current study received antipsychotics. Given that antipsychotics may impact CT in patients with schizophrenia, this is a particularly important confounding factor in the current study as patients with TRS received clozapine that seems to have a unique mechanism of action different than first-line treatment received by patients with NTRS (van Erp et al., 2018, Ansell et al., 2015, Nesvåg et al., 2008). As such, the current findings may reflect the ramifications of antipsychotics treatment rather than the pathophysiology of schizophrenia. However, it should be noted that the classifier brain regions were not supposed to be vulnerable to antipsychotics (Zugman et al., 2013, Lesh et al., 2015). Instead, it was noted that antipsychotics-naïve patients presented with thin CT in these brain regions (Nelson et al., 2020). Indeed, in the current study, CT values in the brain regions serving as a classifier were not correlated with chlorpromazine equivalent (all *p* > 0.1). Taken together, these findings suggest that it is unlikely that the current findings are derived from antipsychotic treatment.

We are unaware of any study that aimed to examine the dimensionality of TRS and NTRS using neuroimaging data. Thus, the novelty and significance of the current study are on the exchangeability of classifiers, which suggests the continuity of two conditions that were previously thought distinct. However, some parts of the current findings are in line with prior studies. For example, we demonstrated that the PT was a classifier for NTRS. The PT is an upper side of the superior temporal sulcus of which abnormal CT was frequently reported in patients with schizophrenia (van Erp et al., 2018). Additionally, STS was proposed as the neural basis of auditory hallucination (Walton et al., 2017, Cachia et al., 2008). Abnormal insular CT (van Erp et al., 2018), volume (Takahashi et al., 2009), and functional connectivity were often reported in this population (Manoliu et al., 2014). Further, the associations between IFG and a wide variety of symptoms, such as deficits in executive function (Jirsaraie et al., 2018), language (Jeong et al., 2009), and semantic processing (Jeong et al., 2009) were also reported. Taken together the reported classifiers agree with previous CT reports in patients with schizophrenia (van Erp et al., 2018, Walton et al., 2017, Cachia et al., 2008, Takahashi et al., 2009, Manoliu et al., 2014, Jirsaraie et al., 2018, Jeong et al., 2009, Kubicki et al., 2011). Despite these findings from prior studies, CT values in these brain regions were not associated with the PANSS scores in the current sample (see Supplementary Information). There are some potential explanations for the lack of association. First, these brain regions may be associated with the cognitive component but not with PANSS score; a sum of a variety of symptoms. Second, these brain regions may represent the categorical effect of diagnosis rather than the dimensional impact of symptoms. As the brain-behavior association is not a scope of the current study, future studies are expected to fill this gap.

It should be noted that the current study assumed a specific subset of brain regions serve as classifiers for both TRS and NTRS, which is based on some prior studies that reported sparseness in classifying psychiatric diseases (Yahata et al., 2016, Takahashi et al., 2009). Given that the prior studies showed diffusely thinner CT in both TRS and NTRS compared with HC, this assumption seems to be contradictory. However, the statistical difference in the CT values does not always mean the dearth of overlap in the CT values. In the case where the CT value distributions largely overlap between TRS and NTRS, it is not possible to classify the TRS from NTRS even if diffuse brain regions show the difference. In contrast, if the difference in CT values appeared in only sparse brain regions, TRS can be successfully classified from NTRS as long as the distribution of the two conditions does not overlap. In line with this notion, our preliminary analysis using ridge logistic regression, which does not assume sparseness, did not show high classification performance (see Supplementary Information). On the other hand, in line with the prior studies, the group comparison showed a diffuse thin CT pattern in TRS and NTRS compared with HC in the current sample. These results from supplementary analyses suggest that the brain regions do not exist without overlapping in CT value distributions between TRS and NTRS.

The current study has several limitations. First, although we applied a well-established harmonization method (Fortin et al., 2018, Johnson et al., 2007), we integrated data from two sites. In addition to the difference in scan parameters, there may be a difference in race and ethnicity. The cross-validation framework would imply that the current findings were robust to these confounding factors. However, because of the sample size, it was not statistically possible to repeat the same analyses in the CAMH and Komagino datasets, separately. To further examine the generalizability of the current findings, a future study with larger sample size is expected to conduct external validation. Second, although we examined the impact of antipsychotics by standardizing the amount with chlorpromazine equivalent. However, the effects of first-line antipsychotics versus clozapine may be different (Breiman, 1996). Although we are not aware of accepted protocols other than chlorpromazine equivalent, future research is expected to quantify the impact of antipsychotics by types. Third, we determined the importance of brain regions based on the frequency of being selected by the L1 regularization method during the 10-fold CV. However, significant results do not always mean that significant regions convey important information regarding neural underpinnings of TRS or NTRS, instead indicate the significance of within-sample reliability. Prior studies showed that coefficients from machine learning methods, even from the linear ones, are not straightforward to be interpreted due to noise in the decoding process (Haufe et al., 2014, Kia et al., 2016), thus required to be transformed (Haufe et al., 2014). Because the current study did not transform the coefficients, the current results need to be treated with caution. Fourth, as we demonstrated commonality in classifiers in both TRS and NTRS, it raises the question of whether the shared classifier explains the lack of drop in the classification performance when the classifiers were swapped. In other words, PT, which was identified as a classifier in both TRS and NTRS, may contribute to the classification performance in large part. Although this assumption is intriguing, conducting the same analyses excluding PT does not get the point but loses the essence of the pathophysiology of schizophrenia because the current study suggests that PT is a critical brain region in both TRS and NTRS. Besides, even if the PT explains a lot of classification performance, it does not affect the conclusion of the current study. Thus, future study is expected to see the replicability of the importance of PT in an independent sample.

It is striking that two groups that showed a distinct pattern in treatment response (i.e., TRS and NTRS) actually shared the abnormal cortical features. The current finding may appear inconsistent with two influential hypotheses for schizophrenia, namely, predominantly-glutamate and predominantly-dopamine abnormal systems for TRS and NTRS, respectively. However, from a neurochemical perspective, these two systems should not be considered categorically distinct. More specifically, an abnormal glutamate system in the cortex leads to an excessive dopamine level in the striatum downstream (Javitt, 2007, McCutcheon et al., 2020). Thus, abnormality in glutamate systems would add an extra layer of abnormality instead of providing distinct abnormality to the dopamine system. As a matter of fact, prior ^1^H-MRS studies demonstrated that both patients with TRS and patients with NTRS had abnormally elevated glutamate levels in the anterior cingulate cortex (Tarumi et al., 2020, Merritt et al., 2016). Besides, patients with TRS presented with a greater deviation of glutamate level from the norm than NTRS (Demjaha et al., 2014) and remained elevated glutamate level after treatment (Merritt et al., 2021). In summary, we speculate that glutamate- and dopamine-predominant abnormalities converge at the cortical level, retaining the difference in the point where abnormalities start. Future research is expected to examine the relationship between the multiple aberrant neurotransmitter systems and cortical features.

## 5 Conclusion

The present CT analysis indicates that TRS and NTRS share certain similar characteristics at the cortical level, and TRS presents exclusive differences in the right aSTS and right IOFC. The results may indicate that TRS may be a more advanced form of NTRS. However, TRS could be a ‘comorbid’ entity with some CT commonalities with NTRS, and different medication treatment (clozapine for TRS versus first-line antipsychotic for NTRS) may contribute to CT differences. The results support the need of large longitudinal studies to clarify the nature of the differences between TRS and NTRS.

## Author contributions

Conceptualization: TI, YN, YYA, AGG, and SN. Data curation: YI, RT, ST, EP, SH, FC, JK, KM, PG, HU, and GR. Formal analysis: TI and YYA. Writing - original draft: TI and YYA. Writing - review & editing: YN, MM, AGG, and SN.

## Declaration of Competing Interest

The authors declare that they have no known competing financial interests or personal relationships that could have appeared to influence the work reported in this paper.
