# Supplementary material for: Dimensional distribution of cortical abnormality across antipsychotics treatment-resistant and responsive schizophrenia
Source: Neuroimage Clin. 2021 Oct 7;32:102852. doi: 10.1016/j.nicl.2021.102852 (PMC8527893; doi:10.1016/j.nicl.2021.102852)
Supplement: Supplementary data 1 [file mmc1.docx]

**Supplementary Information**

**Title**

Dimensional distribution of cortical abnormality across antipsychotics treatment-resistant and responsive schizophrenia

**Running title**

TRS shares CT features with NTRS

**Authors**

Takashi Itahashi, Ph.D.^1^, Yoshihiro Noda, M.D., Ph.D., M.B.A.^2^, Yusuke Iwata, M.D., Ph.D.^3^, Ryosuke Tarumi, M.D., Ph.D.^2^, Sakiko Tsugawa, M.D.^2^, Eric Plitman, Ph.D.^5^, Shiori Honda, M.Sc^2^, Fernando Caravaggio, Ph.D.^4, 5^, Julia Kim, B.Sc.^4, 5^, Karin Matsushita, B.A.^2^, Philip Gerretsen, M.D., Ph.D.^4, 5^, Hiroyuki Uchida, M.D. Ph.D.^2, 4^, Gary Remington, M.D., Ph.D.^4, 5^, Masaru Mimura, M.D., Ph.D.^2^, Yuta Y. Aoki, M.D., Ph.D.^1*^, Ariel Graff-Guerrero, M.D., Ph.D.^4, 5*^, Shinichiro Nakajima, M.D., Ph.D.^2, 4*^

**Affiliations**

1 Medical Institute of Developmental Disabilities Research, Showa University, Tokyo, Japan

2 Department of Neuropsychiatry, Keio University School of Medicine, Tokyo, Japan

3 Department of Neuropsychiatry, University of Yamanashi Faculty of Medicine, Yamanashi, Japan

4 Brain Health Imaging Centre, Centre for Addiction and Mental Health (CAMH), Toronto, Ontario, Canada

5 Department of Psychiatry, University of Toronto, Toronto, Ontario, Canada

***Correspondence:**

Yuta Y. Aoki, MD. Ph.D.

Senior Assistant Professor

Medical Institute of Developmental Disabilities Research, Showa University, 6-11-11 Kita-karasuyama, Setagaya-ku, Tokyo 157-8577, Japan.

Tel: +81-3-5315-9357 (et. 182) Fax: +81-3-5315-9358

E-mail: youyouryuta@gmail.com

Ariel Graff-Guerrero, M.D., Ph.D.

Centre for Addiction and Mental Health

80 Workman Way, 6th Floor

Toronto, ON M6J1H4, Canada

Tel: 416-535-8501 (ext. 34834)

Emails: ariel_graff@yahoo.com.mx; ariel.graff@camh.ca

Shinichiro Nakajima, M.D., Ph.D.

Department of Neuropsychiatry, Keio University School of Medicine, Tokyo, Japan

35 Shinanomachi, Shinjuku-ku, Tokyo, Japan, 160-8582

Phone: +81-3-3353-1211 (ext. 62454) Fax: +81-3-5379-0187

Email: shinichiro_nakajima@hotmail.com

**Construction of classifier for differentiating patients with TRS from patients with NTRS**

To examine whether patients with TRS and those with NTRS have a shared or distinct neural basis, we built a classifier distinguishing the TRS group from the NTRS group using logistic regression with the least absolute shrinkage and selection operator (LASSO) method [1]. We used a 10-fold cross-validation (CV) procedure with an undersampling method in the same manner as the main analyses. We repeated the random sampling procedure ten times in each loop. These procedures, thus, yielded ten classifiers in each loop. Once ten classifiers were trained in each loop, we applied these classifiers to the test dataset and computed the mean classifier output value (diagnostic probability). We considered participants as TRS if their diagnostic probability values were greater than 0.5. We calculated the area under the curve (AUC) as an index for the classification performance. We also computed the accuracy, sensitivity, and specificity.

To examine the statistical significance of the classification performance, we used a permutation test with 1,000 iterations. We permuted the labels of the training dataset and conducted a 10-fold CV with a 10-undersampling procedure. We computed the mean diagnostic probability obtained from the permuted classifiers at each iteration. We considered participants as TRS if their mean diagnostic probability values were higher than 0.5. We then computed the ACU value at each iteration. We then constructed a null distribution of the AUC values. Of note, we constructed a null distribution as the max distribution of the AUC values across three classifiers (i.e., HC-NTRS, HC-TRS, and NTRS-TRS) to control for the multiple comparisons. Statistical significance was set at p < 0.05, one-sided.

As shown in Figure S1, the classifiers differentiated patients with TRS from those with NTRS with an accuracy of 56% and an AUC of 0.56 (p = 0.37 FWE-corrected). These results suggest that, at least in our dataset, the classifier could not distinguish patients with TRS from NTRS successfully.

**Estimation of inter-site differences inside the loop**

In the main analyses, we applied a ComBat harmonization method outside the 10-fold CV, raising the possibility that the classification performance was improved due to information leakage. To confirm the robustness of our findings, we repeated our analyses while applying the ComBat harmonization method inside the 10-fold CV procedure. We computed the mean diagnostic probability and considered participants as TRS or NTRS if their diagnostic probability values were greater than 0.5. We then computed the AUC value and accuracy to investigate the classification performance.

Similar to our main findings, both classifiers could successfully distinguish patients with NTRS or TRS from HCs (Figure S2). The classifiers distinguished patients with NTRS from HCs with an accuracy of 63% with an AUC of 0.70. Sensitivity and specificity were 65% and 61%, respectively. The other set of classifiers differentiated patients with TRS from HCs with an accuracy of 76% with an AUC of 0.85. Sensitivity and specificity were 73% and 78%, respectively. These results support the robustness of our main findings.

**Estimation of inter-site differences inside the loop**

In the main analyses, we applied a ComBat harmonization method outside the 10-fold CV, raising the possibility that the classification performance was improved due to information leakage. To confirm the robustness of our findings, we repeated our analyses while applying the ComBat harmonization method inside the 10-fold CV procedure. We computed the mean diagnostic probability and considered participants as TRS or NTRS if their diagnostic probability values were greater than 0.5. We then computed the AUC value and accuracy to investigate the classification performance.

Similar to our main findings, both classifiers could successfully distinguish patients with NTRS or TRS from HCs (Figure S2). The classifiers distinguished patients with NTRS from HCs with an accuracy of 63% with an AUC of 0.70. Sensitivity and specificity were 65% and 61%, respectively. The other set of classifiers differentiated patients with TRS from HCs with an accuracy of 76% with an AUC of 0.85. Sensitivity and specificity were 73% and 78%, respectively. These results support the robustness of our main findings.

**Estimation of inter-site differences only using healthy controls**

It might be useful to estimate the inter-site differences from HCs if the proportion of patients was different between the two sites. To confirm this possibility, we compared the F-values of the two differently harmonized datasets using mass univariate analysis. One is a dataset corrected for inter-site differences estimated from the whole dataset. The other is a dataset corrected for such differences estimated only from HCs. Of note, we incorporated age, sex, and years of education as biological variables in the ComBat. The harmonized datasets were fed into a one-way analysis of variance (ANOVA) to compute the F-value in each brain region. The similarity between the two datasets was computed using the Pearson correlation coefficient. Statistical significance was set at p < 0.05. As shown in Figure S3, we found that the F-values obtained from the two datasets were statistically significantly correlated (r = 0.97, 95%CI = [0.96, 0.98], p < 0.001). The 95% confidence interval (95%CI) was estimated using the “bootci” function implemented in MATLAB (2020b, Mathworks). This result suggested that, at least in our dataset, estimation of the inter-site differences from HCs did not provide significant advantages.

**Construction of classifiers with nested five-fold cross-validation procedure**

In the main analyses, we used a nested 10-fold cross-validation (CV) procedure. To investigate the robustness of our findings against the CV procedures, we repeated the same analyses with a five-fold CV procedure.

Similar to our main findings, both classifiers distinguished patients with NTRS or TRS from HCs. The classifiers distinguished patients with NTRS from HCs with an accuracy of 66% with an AUC of 0.69. Sensitivity and specificity were 63% and 70%, respectively. The other set of classifiers differentiated patients with TRS from HCs with an accuracy of 78% with an AUC of 0.85. Sensitivity and specificity were 82% and 75%, respectively. These results support that our main findings are robust to the choice of CV procedures.

**Group comparisons between the TRS and NTRS groups**

We performed two-tailed two-sample t-tests to examine the group differences between the TRS and NTRS group in the CT values. Statistical significance was set at q < 0.05 after false discovery rate (FDR) correction [2]. We also computed the standardized effect size estimates (Cohen’s d). Statistical analyses revealed that patients with TRS exhibited reduced CT values in a wide range of brain regions, including the bilateral inferior frontal cortices, anterior and posterior cingulate cortices, and visual cortices (Figure S4).

**Classification analysis with ridge logistic regression**

We observed statistically significant differences in CT values between the TRS and NTRS groups in the univariate analyses. These notions raised the question that if patients with TRS exhibited alterations as a whole-brain pattern compared with the NTRS group, other non-sparse classifiers could distinguish patients with TRS from those with NTRS. To answer this question, we repeated classification analyses with a 10-fold CV using ridge logistic regression. Of note, we used ridge logistic regression rather than a simple logistic regression because the number of features was greater than the number of participants in our dataset. We used the “fitclinear” function implemented in MATLAB (2020b, Mathworks). We tuned the hyperparameter by setting “OptimizeHyperparameters” as “Lambda” with the grid search. The classifiers distinguished patients with TRS from those with NTRS with an accuracy of 59% with an AUC of 0.59. Sensitivity and specificity were 59% and 59%, respectively. Although the classification performance was slightly improved when compared with LASSO, the classification performance was still poor. These findings imply that alterations might be specific to brain regions rather than the whole-brain pattern.

**Associations between the symptom severity and CT values**

To assess the associations between the symptom severity measured by PANSS and CT values identified by the LASSO method, we performed correlation analyses within the patient group. We used Spearman’s rank correlation coefficient as a measure, and statistical significance was set at p < 0.05. In addition, we performed correlational analyses on the TRS and NTRS groups separately.

As shown in Table S2, correlation analyses within the merged patient group revealed weak negative correlations between the symptom severity and CT values: PANSS positive score was negatively correlated with CT values in the left inferior frontal gyrus (IFG) (rs = -0.20, p = 0.04, uncorrected), while PANSS negative score was negatively correlated with CT values in the left anterior insula/IFG (aINS/IFG) (rs = -0.21, p = 0.03, uncorrected). As shown in Table S3, correlation analyses in each patient group revealed that PANSS negative symptom subscale score was negatively correlated with the CT values in the left aINS/IFG (rs = -0.27, p = 0.04, uncorrected) in the TRS group.

We also performed correlation analysis to assess the association between CPZ equivalent and CT values identified by the LASSO method. Correlation analyses revealed no statistically significant associations between the CPZ equivalent and CT values (all p > 0.07; see Table S2 and Table S3, for details).

**Supplementary References**

1. Tibshirani, R., 1996. Regression Shrinkage and Selection Via the Lasso. Journal of the Royal Statistical Society: Series B (Methodological). https://doi.org/10.1111/j.2517-6161.1996.tb02080.x

2. Storey, J.D., 2002. A direct approach to false discovery rates. J. R. Stat. Soc. Series B Stat. Methodol. 64, 479–498.

**Supplementary Figures**


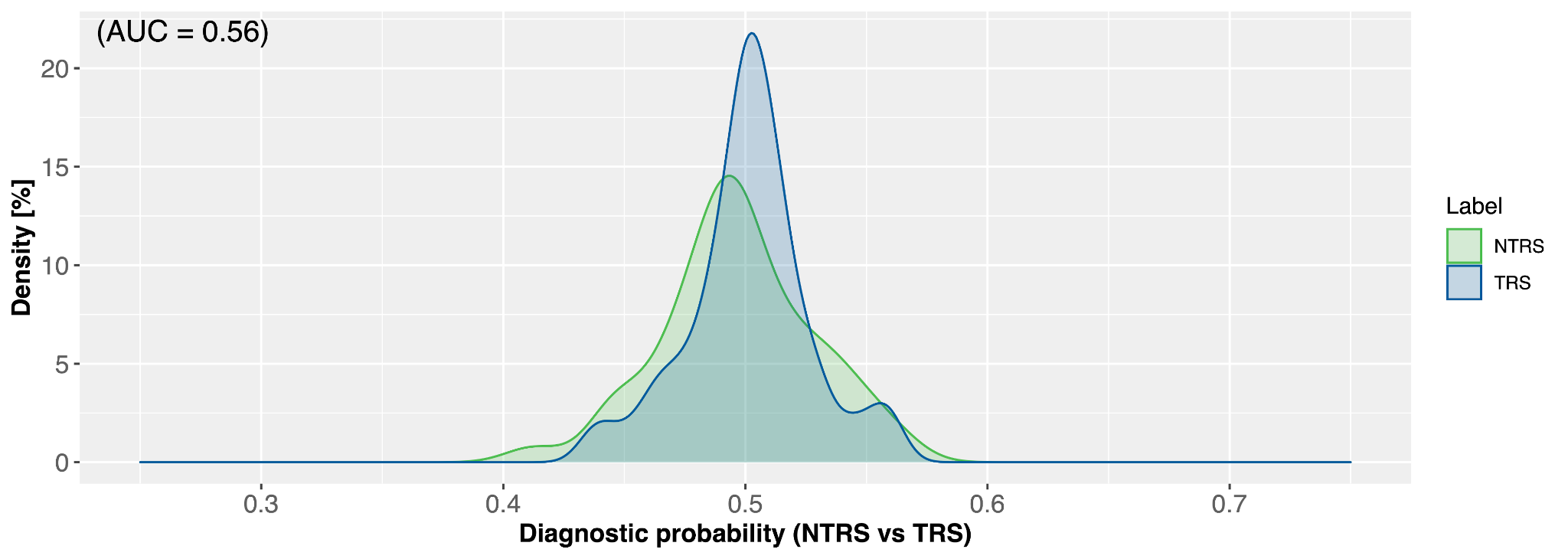


**Figure S1. The classification performance of the classifier for differentiating patients with TRS from those with NTRS.**

The classification performance was measured by the area under the curve (AUC). The classifier distinguished patients with treatment-resistant schizophrenia (TRS) from non-treatment-resistant schizophrenia (NTRS) with an accuracy of 56% with an AUC of 0.56 (FWE-corrected, p = 0.36).


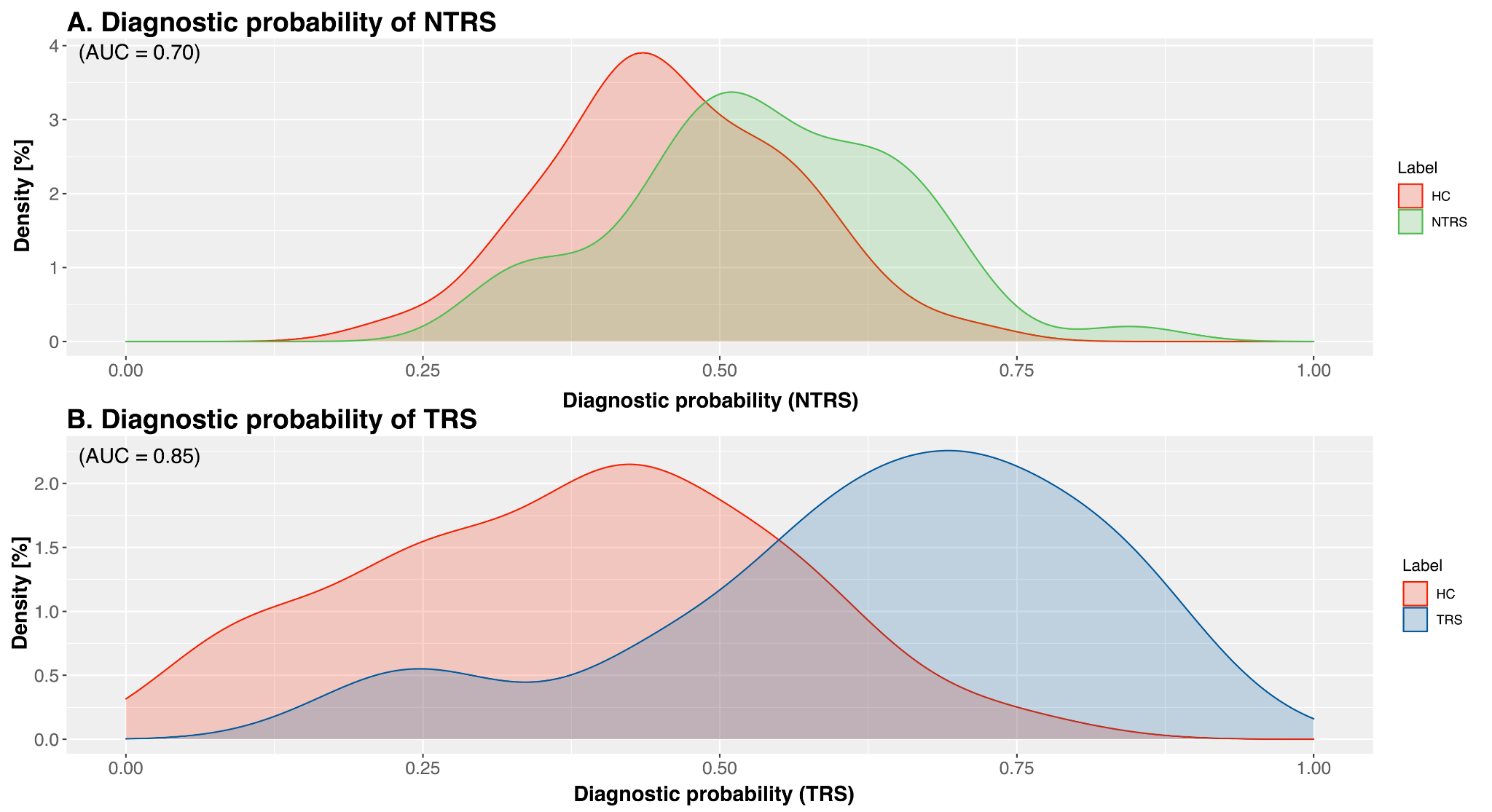


**Figure S2. The classification performance of the classifier for NTRS and TRS when applying ComBat harmonization inside the 10-fold cross-validation (CV).**

(A) The diagnostic probability distribution for non-treatment-resistant schizophrenia (NTRS) and (B) diagnostic probability distribution for treatment-resistant schizophrenia (TRS). We computed the area under the curve (AUC).


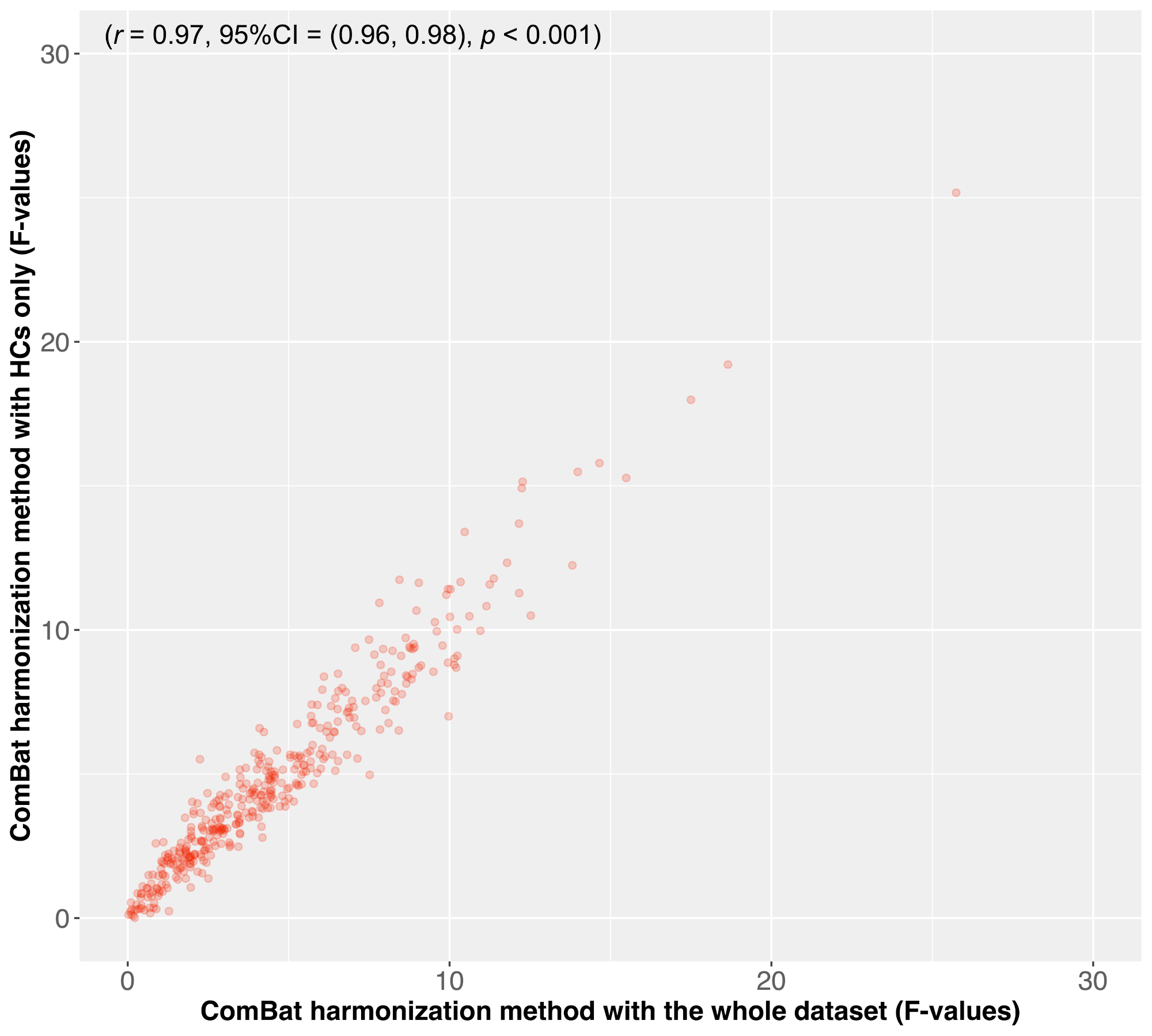


**Figure S3. Scatter plots of F-values of harmonized data using the whole dataset versus harmonized data only from the healthy controls (HCs).** Pearson correlation coefficient was computed to examined their similarity. The Pearson correlation coefficient of F-values from both harmonized datasets was 0.97 (95% confidence interval (95%CI) = (0.96, 0.98), p < 0.001).


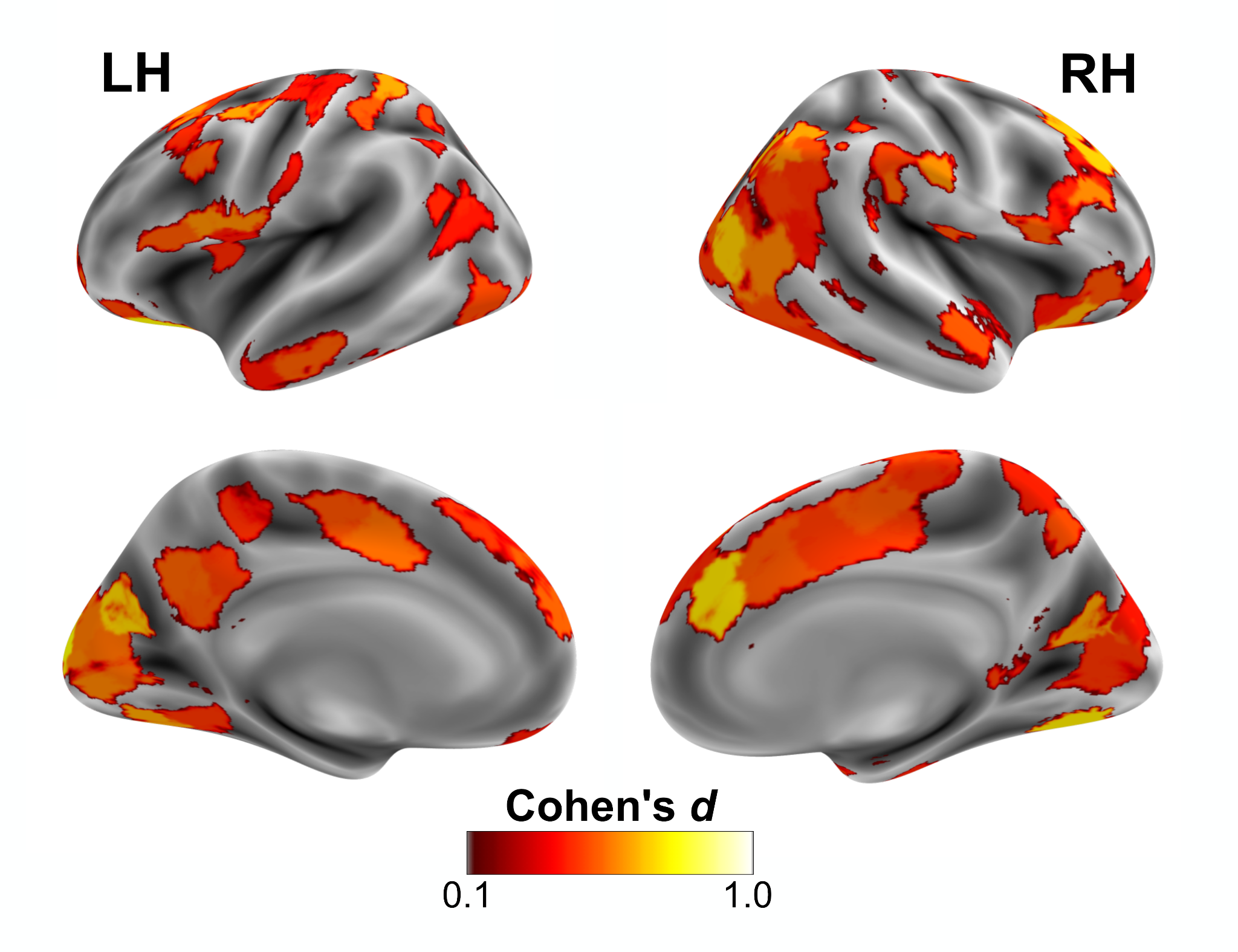
**Figure S4. Comparisons between the TRS and NTRS groups.**

Two-sample two-tailed t-tests with false discovery rate (FDR) correction revealed that, compared with the non-treatment-resistant schizophrenia (NTRS) group, the treatment-resistant schizophrenia (TRS) group exhibited thinner cortical thickness values in several brain regions.
