# Supplementary material for: Dimensional distribution of cortical abnormality across antipsychotics treatment-resistant and responsive schizophrenia
Source: Neuroimage Clin. 2021 Oct 7;32:102852. doi: 10.1016/j.nicl.2021.102852 (PMC8527893; doi:10.1016/j.nicl.2021.102852)
Supplement: Supplementary data 2 [file mmc2.docx]

**Table S2. Demographic information for two sites**

|  | HC | | Statistics | | | NTRS | | Statistics | | | TRS | | Statistics | | |
| --- | --- | --- | --- | --- | --- | --- | --- | --- | --- | --- | --- | --- | --- | --- | --- |
|  | Komagino | CAMH | Statistics | *df* | *P*-values | Komagino | CAHM | Statistics | *df* | *P*-values | Komagino | CAMH | Statistics | *df* | *P*-values |
| N (Female) | 27 (16) | 25 (7) | 5.1 | - | 0.02 | 29 (17) | 17 (4) | 5.3 | - | 0.02 | 21 (11) | 43 (9) | 6.5 | - | 0.01 |
| Age, year (mean ± SD) | 42.7 ± 11.5 | 40.2 ± 13.2 | 0.7 | 50 | 0.47 | 41.9 ± 13.2 | 45.5 ± 13.5 | -0.9 | 44 | 0.38 | 42.2 ± 10.8 | 43.1 ± 12.9 | -0.25 | 62 | 0.80 |
| Duration of illness, year | - | - | - | - | - | 15.6 ± 12.5 | 20.6 ± 11.3 | -1.3 | 44 | 0.19 | 17.9 ± 10.5 | 19.1 ± 12.4 | -0.38 | 57 | 0.70 |
| PANSS total score | - | - | - | - | - | 49.2 ± 14.1 | 58.9 ± 8.5 | -2.5 | 43 | 0.02 | 108.7 ± 18.5 | 70.2 ± 17.0 | 8.1 | 58 | < 0.01 |
| Positive symptom subscale | - | - | - | - | - | 9.8 ± 2.7 | 11.4 ± 2.3 | -2 | 43 | 0.05 | 26.5 ± 4.5 | 17.2 ± 6.2 | 6.0 | 58 | < 0.01 |
| Negative symptom subscale | - | - | - | - | - | 14.1 ± 6.0 | 16.5 ± 3.4 | -1.5 | 43 | 0.15 | 30.0 ± 5.9 | 18.7 ± 4.7 | 8.2 | 58 | < 0.01 |
| General psychopathology subscale | - | - | - | - | - | 25.3 ± 6.8 | 31.1 ± 4.2 | -3.1 | 43 | < 0.01 | 52.2 ± 10.6 | 34.5 ± 8.9 | 6.9 | 58 | < 0.01 |
| CGI-S | - | - | - | - | - | 2.3 ± 0.8 | 1.7 ± 0.5 | 3.0 | 42 | < 0.01 | 5.1 ± 0.4 | 2.4 ± 0.5 | 21.5 | 58 | < 0.01 |
| CPZ equivalent daily dose, (mg) | - | - | - | - | - | 401.2 ± 224.5 | 417.9 ± 183.1 | -0.3 | 44 | 0.80 | 958.9 ± 522.5 | 550.5 ± 238.3 | 4.3 | 61 | < 0.01 |
| **Abbreviations:** CGI-S: Clinical Global Impression Severity Scale, CPZ: chlorpromazine, HC: healthy control, NTRS: non-treatment-resistant schizophrenia, PANSS: Positive and Negative Symptom Scale, SD: standard deviation, TRS: treatment-resistant schizophrenia | | | | | | | | | | | | | | | |
