# Supplementary material for: Dimensional distribution of cortical abnormality across antipsychotics treatment-resistant and responsive schizophrenia
Source: Neuroimage Clin. 2021 Oct 7;32:102852. doi: 10.1016/j.nicl.2021.102852 (PMC8527893; doi:10.1016/j.nicl.2021.102852)
Supplement: Supplementary data 3 [file mmc3.docx]

**Table S2. Results of correlation analyses within the patient group.**

| **Brain regions** | **PANSS score** | | | | **CPZ equivalent daily dose, (mg)** |
| --- | --- | --- | --- | --- | --- |
|  | **Total score** | **Positive symptom subscale** | **Negative symptom subscale** | **General psychopathology subscale** |  |
| Left PT | -0.10 (0.33) | -0.13 (0.20) | -0.05 (0.64) | -0.11 (0.26) | -0.00 (0.97) |
| Left aINS/IFG | -0.17 (0.07) | -0.12 (0.22) | **-0.21 (0.03)** | -0.18 (0.07) | -0.04 (0.66) |
| Left IFG | **-0.19 (0.04)** | **-0.20 (0.04)** | -0.16 (0.11) | -0.18 (0.07) | -0.14 (0.16) |
| Left SMG | 0.07 (0.50) | 0.11 (0.25) | -0.00 (0.99) | 0.07 (0.48) | 0.08 (0.42) |
| Right aSTS | -0.14 (0.17) | -0.12 (0.23) | -0.18 (0.07) | -0.12 (0.23) | -0.02 (0.84) |
| Right lOFC | -0.11 (0.25) | -0.15 (0.12) | -0.09 (0.35) | -0.09 (0.37) | -0.01 (0.88) |
| **Abbreviations:** aINS: anterior insula, aSTS: anterior superior temporal sulcus, CPZ equivalent: Chlorpromazine equivalent, IFG: inferior frontal gyrus, lOFC: lateral orbitofrontal cortex, SMG: supramarginal gyrus.  Bold numbers represent *p* < 0.05, uncorrected. | | | | | |
