# Supplementary material for: Dimensional distribution of cortical abnormality across antipsychotics treatment-resistant and responsive schizophrenia
Source: Neuroimage Clin. 2021 Oct 7;32:102852. doi: 10.1016/j.nicl.2021.102852 (PMC8527893; doi:10.1016/j.nicl.2021.102852)
Supplement: Supplementary data 4 [file mmc4.docx]

**Table S3. Results of correlation analyses in each patient group**

|  | **NTRS** | | | | | **TRS** | | | | |
| --- | --- | --- | --- | --- | --- | --- | --- | --- | --- | --- |
| **Brain regions** | **PANSS score** | | | | **CPZ equivalent daily dose, (mg)** | **PANSS score** | | | | **CPZ equivalent daily dose, (mg)** |
|  | **Total score** | **Positive symptom subscale** | **Negative symptom subscale** | **General psychopathology subscale** |  | **Total score** | **Positive symptom subscale** | **Negative symptom subscale** | **General psychopathology subscale** |  |
| Left PT | -0.08 (0.60) | -0.16 (0.29) | -0.03 (0.86) | -0.07 (0.67) | -0.01 (0.95) | -0.04 (0.75) | -0.09 (0.50) | 0.03 (0.84) | -0.08 (0.54) | 0.07 (0.61) |
| Left aINS/IFG | -0.12 (0.43) | -0.06 (0.70) | -0.19 (0.22) | -0.02 (0.87) | 0.06 (0.67) | -0.23 (0.08) | -0.18 (0.17) | -0.23 (0.08) | **-0.27 (0.04)** | -0.09 (0.46) |
| Left IFG | -0.07 (0.63) | -0.00 (0.98) | -0.09 (0.55) | -0.03 (0.82) | 0.05 (0.74) | -0.02 (0.90) | -0.03 (0.83) | -0.00 (0.98) | -0.03 (0.82) | -0.03 (0.81) |
| Left SMG | 0.00 (0.99) | 0.13 (0.41) | -0.07 (0.65) | -0.03 (0.83) | 0.16 (0.28) | 0.00 (0.85) | 0.00 (0.98) | -0.06 (0.64) | 0.08 (0.54) | -0.04 (0.73) |
| Right aSTS | -0.06 (0.6 | -0.09 (0.56) | 0.01 (0.96) | -0.08 (0.61) | 0.17 (0.25) | 0.03 (0.80) | 0.08 (0.52) | -0.12 (0.37) | 0.06 (0.67) | 0.02 (0.91) |
| Right lOFC | 0.11 (0.48) | 0.05 (0.76) | 0.11 ( | 0.13 (0.41) | 0.27 (0.07) | 0.06 (0.63) | 0.05 (0.72) | 0.06 (0.67) | 0.06 (0.66) | 0.06 (0.64) |
| **Abbreviations:** aINS: anterior insula, aSTS: anterior superior temporal sulcus, CPZ equivalent: Chlorpromazine equivalent, IFG: inferior frontal gyrus, lOFC: lateral orbitofrontal cortex, SMG: supramarginal gyrus. Bold numbers represent *p* < 0.05, uncorrected. | | | | | | | | | | |
